# Case of a Fatal Ulceration of the Bladder, Occasioned by a Caries of the Os Pubis; with an Account of the Appearances on Dissection

**Published:** 1782

**Authors:** 


II. *Case of a fatal ulceration of the bladder, occasioned by a caries of the os pubis; with an account of the appearances on dissection. By* Mr. Edward Ford, *Surgeon to the Westminster General Dispensary. Communicated in a letter to* Samuel Foart Simmons, M.D. F.R.S. *and by him to the Society. Read* Feb. 4, 1782.

MARY Hudson, the patient of whose case you have requested me to give you an account, was a healthy girl 'till about the age of fourteen, when she began to complain of great pain in making water, and voided several small stones with her urine, which was generally purulent and bloody. She could not ascertain a more probable cause of her disorder, than that she formerly had received a blow upon the pubis bis. Either the poverty of her relations, or her own delicacy, prevented an immediate application for relief. Seven or eight months had elapsed before ſhe made known to her friends the nature of her diſeaſe, and as it did not then appear alarming, they were not immediately urgent for medical aſſiſtance; but they were ſoon after convinced of the imminent danger of her ſituation. It was at this period of the diſeaſe that I firſt ſaw her. She was then in a very emaciated ſtate, and beſides the local ſymptoms abovementioned, was troubled with an hectic cough, univerſal languor and debility, nocturnal ſweats, and every concomitant of an abſorption of matter. Theſe ſymptoms, ſoon after, terminated in death. Being deſirous of examining the ſtate of the bladder, as the ſymptoms of the caſe indicated ſome extraordinary diſeaſe there, I procured leave to open the dead body.

The external view of the parts afforded nothing preternatural. The abdomen was opened in the uſual manner, but there was not the leaſt morbid appearance in any of the contained viſcera. The bladder alone ſeemed ſtrongly contracted and thickened, a circumſtance common to thoſe who have from diſeaſe a habit of expelling their urine before it is collected to a confiderable quantity. Upon introducing a catheter into the bladder, feveral fmall ftones were felt, and a little purulent urine was evacuated. I now fufpended my enquiry till the bladder with the parts adjacent were taken from the body. In the courfe of this diffection, in feparating the bladder from its connexion with the fymphyfis of the os pubis, the point of the knife paffed into a cavity of that bone, from which iffued a fpoonful or more of matter. This cavity in the os pubis was found to be adapted to, and exactly to communicate with an ulcer in the bladder. When the parts were removed, the whole fubftance of the os pubis was found to be carious. Anteriorly it was defended only by a ligamentous covering, and behind was the cavity juft now fpoken of, communicating with the bladder, and containing little pieces of bone that were almoft wholly incrufted with a fabulous matter. There was alfo a hollow leading down from hence to the tuberofity of the ifchion on the left fide, this part of the bone being alfo carious, and feveral bits which were feparated from it, likewife covered with a fimilar concretion.

From

From this view of the parts it was evident that an inſtrument introduced into the bladder might have paſſed into the centre of the bone; and as the matter diſcharged by the meatus urinarius ſeemed to have its ſource from this caries, it was fortunate that the girl was not ſearched for the ſtone, as any operation which the ſuppoſed exiſtence of calculi might have rendered adviſeable, would have ſuffered a diſgrace from its unavoidable ill ſucceſs.

The ſtones found in the bladder were evidently incruſtations upon nuclei of exfoliated laminæ from the bone which had penetrated into the bladder.

The primary cauſe of all this miſchief ſeems to have been the contuſion, or perhaps fracture of the bone; but it is difficult to explain why the corroſive diſcharge which had ſo much affected the iſchion, did not find a paſſage from thence to the inſide of the thigh, and form a ſuppuration there, ſince that part of the bone to which the two heads of the triceps muſcle are attached, was almoſt entirely deſtroyed. The patient had, indeed, ſome time before her death complained of a pain on the inſide of her thigh, to which topical remedies had been applied, but we could not find there any marks of an approaching ſuppuration.

